# CRAC channel activity pulsates during cytosolic Ca^2+^ oscillations

**DOI:** 10.1016/j.jbc.2025.108519

**Published:** 2025-04-23

**Authors:** Yu-Ping Lin, Erica Scappini, Gary R. Mirams, Charles J. Tucker, Anant B. Parekh

**Affiliations:** 1 Laboratory of Signal Transduction, National Institute of Environmental Health Sciences, National Institutes of Health, Durham, North Carolina, USA; 2 Centre for Mathematical Medicine and Biology, School of Mathematical Sciences, University Park, University of Nottingham, Nottingham, UK

**Keywords:** Ca^2+^ signaling, Orai1, Ca^2+^ oscillations, receptors, ion channels

## Abstract

Intracellular Ca^2+^ ions are used as second messengers throughout the phylogenetic tree. They are indispensable for diverse biological processes ranging from fertilization to cell death. In Metazoans, signaling information is conveyed *via* the amplitude, frequency, and spatial profile of cytosolic Ca^2+^ oscillations. In non-excitable cells, these oscillations generally arise from regenerative release of Ca^2+^ from inositol 1,4,5-trisphosphate (InsP_3_)-sensitive intracellular stores, which are refilled by entry of Ca^2+^ through Ca^2+^ release-activated Ca^2+^ (CRAC) channels in the plasma membrane. However, the precise contribution of these store-operated CRAC channels to Ca^2+^ oscillations has remained controversial for decades. One view proposes that CRAC channels remain open throughout stimulation, functioning as the pacemaker in setting Ca^2+^ oscillation frequency. An alternative hypothesis is that channel activity oscillates in parallel with InsP_3_-driven regenerative Ca^2+^ release. Here, by tethering a genetically encoded Ca^2+^ indicator to the pore-forming subunit of the CRAC channel, Orai1, we distinguish between these hypotheses and demonstrate that CRAC channel activity fluctuates in phase with cytosolic Ca^2+^ oscillations during physiological levels of stimulation. We also find that spatially distinct CRAC channel clusters fire in a coordinated manner, revealing that CRAC channels are not independent units but might function in a synchronized manner to provide pulses of Ca^2+^ signal at the same time.

Cytosolic Ca^2+^ oscillations are generated in virtually all cell types. Throughout the life history of the cell, they activate fundamental cellular responses that include exocytosis, metabolism, cell growth and differentiation, and apoptosis (1, 2, 3). Such processes are more effectively activated by Ca^2+^ oscillations than sustained Ca^2+^ rises of similar amplitude or temporal average (4, 5, 6). Indeed, different Ca^2+^-dependent transcription factors are fine-tuned to particular periodicities of Ca^2+^ oscillations in T cells (6), suggesting that cellular functions are regulated by different Ca^2+^ oscillation frequencies. This has given rise to the concept of amplitude and frequency modulation, wherein different responses are activated by distinct amplitudes and frequencies of the oscillations. This concept of signal decoding is now thought to apply to second messengers in general (7, 8).

In electrically non-excitable cells, Ca^2+^ oscillations arise following sub-maximal stimulation of cell-surface receptors that increase levels of the intracellular second messenger inositol trisphosphate (InsP_3_) (1). InsP_3_ binds to and opens large conductance Ca^2+^-permeable ion channels in the endoplasmic reticulum (ER) membrane, resulting in rapid release of stored Ca^2+^ into the cytosol. Some of the cytosolic Ca^2+^ is re-sequestered into stores but a significant fraction is exported out of the cell by plasma membrane Ca^2+^ ATPases (1, 9). Therefore, Ca^2+^ stores must be replenished by entry of extracellular Ca^2+^ through store-operated CRAC channels in the plasma membrane for subsequent waves of Ca^2+^ release to occur (10). Various mechanisms have been proposed to explain oscillatory Ca^2+^ signals including Ca^2+^-dependent activation and inhibition of InsP_3_ receptors, Ca^2+^ regulation of InsP_3_ production, oscillations of intracellular InsP_3_, and mobilization of a second Ca^2+^ pool through Ca^2+^-induced Ca^2+^ release (1, 11). However, a central requirement of all these models is that Ca^2+^ stores are replenished by entry of Ca^2+^ through CRAC channels in the plasma membrane.

CRAC channels are hexamers of plasmalemmal Orai proteins that open following depletion of the ER Ca^2+^ store (12, 13, 14). Their activation is initiated when a fall in ER Ca^2+^ triggers a conformational change in stromal interaction molecules one and 2 (STIM1 and STIM2)—single-pass proteins in the ER membrane (12, 13, 14). This change exposes a domain in STIM1/2 that binds to the cytosolic C-terminus of Orai in regions where the ER is juxtaposed against the plasma membrane (12, 13, 14). STIM1/2 binding opens CRAC channels, allowing Ca^2+^ flux into the cytosol to replenish Ca^2+^ stores and maintain oscillations. Indeed, Ca^2+^ oscillations run down when cells are stimulated in the absence of external Ca^2+^, in the presence of CRAC channel inhibitors, or when Orai1 channels are knocked down (15).

Although the necessity of CRAC channels for oscillatory Ca^2+^ signals is well established, their specific role in regulating the cycle is controversial. One model posits that the channels remain open throughout the oscillatory response, loading the stores with Ca^2+^ and sensitizing the InsP_3_ receptor to ambient levels of InsP_3_ and cytosolic Ca^2+^ (Fig. 1*A*) (16, 17, 18). In this case, CRAC channel-driven store reloading would determine the period between oscillations.

**Figure 1:**
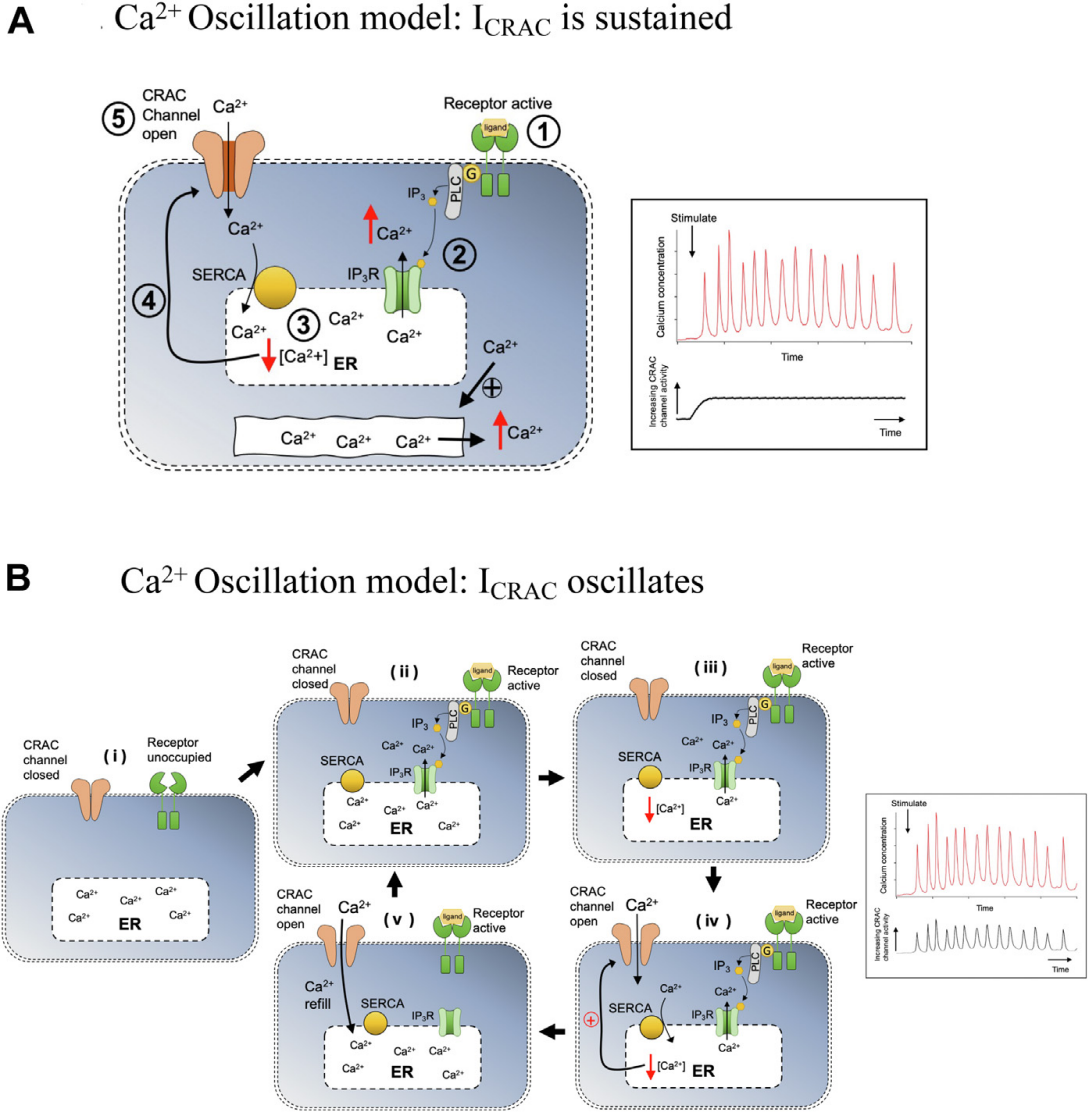
**Cartoons illustrate proposed models for CRAC channel involvement in cytosolic Ca^2+^ oscillations**. *A*, CRAC channels remain open throughout the oscillatory cycle. Receptor engagement (1) leads to the production of InsP_3_ through stimulation of phospholipase C (PLC) *via* a heterotrimeric GTP-binding protein (G). InsP_3_ releases Ca^2+^ from an ER Ca^2+^ store (2), causing a drop in ER Ca^2+^ content (3). *Via* STIM proteins, store depletion (4) then leads to opening of CRAC channels in the plasma membrane (5). Versions of this model include release of ER Ca^2+^ triggering further release from a second Ca^2+^ store *via* Ca^2+^-induced Ca^2+^ release, CRAC channels accelerating reloading of the first ER Ca^2+^ store to trigger Ca^2+^ release by luminal Ca^2+^-dependent regulation of InsP_3_ receptors, and Ca^2+^ flux through the channels sensitizing InsP_3_ receptors to ambient InsP_3_. This model proposes that CRAC channel activity is sustained during an oscillatory response (*right panel*). *B*, CRAC channels activate and deactivate during the oscillatory cycle. In a resting cell (i), receptors are unoccupied, ER is replete with Ca^2+^ and CRAC channels are closed. Upon stimulation (ii), activated receptors increase InsP_3_ levels and ER Ca^2+^ content falls (iii). This leads to store depletion and opening of CRAC channels (iv). Ca^2+^ entry through CRAC channels refills the ER Ca^2+^ store *via* SERCA-dependent Ca^2+^ uptake (v). Store refilling deactivates CRAC channels, and mechanisms that desensitize plasma membrane receptors or InsP_3_ receptors cause cessation of Ca^2+^ release. Following recovery from desensitization, the cell enters phase (ii) again and the cycle repeats. This model proposes that CRAC channel activity fluctuates with each oscillation (*right panel*).

An alternative view is that InsP_3_-driven Ca^2+^ release leads to the opening of CRAC channels, which provide sufficient Ca^2+^ to replenish the stores. As the stores refill, CRAC channels deactivate until they are required to open again following the next cycle of Ca^2+^ release (19, 20). In this model, CRAC channel activity fluctuates during cytosolic Ca^2+^ oscillations (Fig. 1*B*).

Because these two models have markedly different implications for how downstream targets translate Ca^2+^ signals into biological outputs, we sought to determine which was relevant in non-excitable mast cells. Using a genetically encoded Ca^2+^ indicator attached to the N-terminus of Orai1, we find that CRAC channel activity fluctuates in phase with cytosolic Ca^2+^ oscillations evoked by a physiological agonist. Our data provide direct evidence to support the hypothesis that CRAC channel activity oscillates largely in phase with regenerative Ca^2+^ release from the ER.

## Results

### Orai1-GECO fluorescence reflects CRAC channel activity

**Figure 2:**
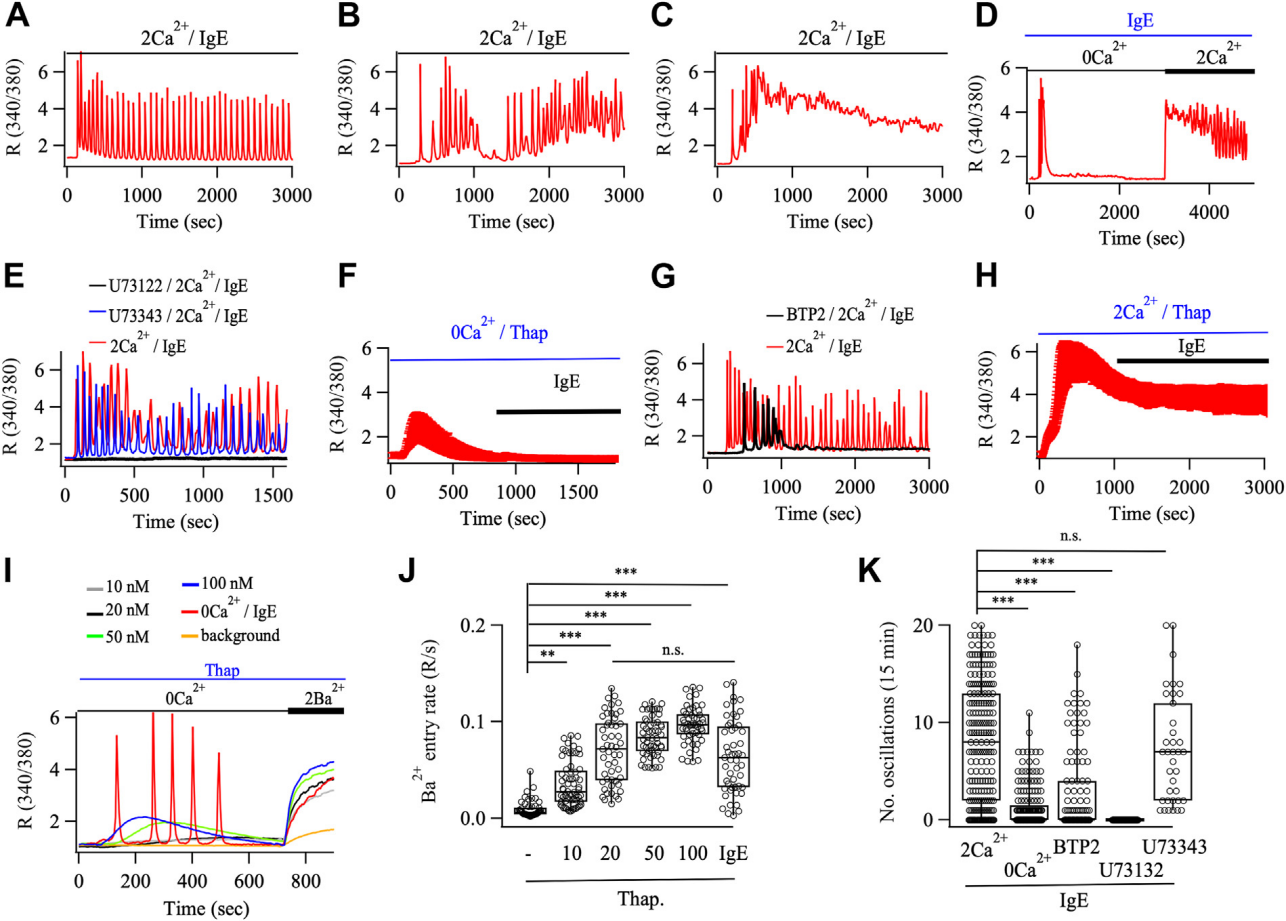
**IgE evokes cytosolic Ca^2+^ oscillations in RBL-2H3 mast cells.***A–C*, three types of response were observed. *A*, the majority of cells responded to IgE by generating numerous baseline Ca^2+^ oscillations. *B*, some cells showed Ca^2+^ oscillations on a gradually rising baseline. *C*, in a minority of cells, a few Ca^2+^ oscillations were followed by a sustained Ca^2+^ plateau. *D*, Ca^2+^ oscillations run down quickly in Ca^2+^-free solution but gradually reappear within 15 min in ∼50% of cells upon readmission of external Ca^2+^. *E*, IgE-evoked Ca^2+^ oscillations are abolished by the phospholipase C inhibitor U73122 (10 μM) but not by the inactive analogue U73343 (10 μM). *F*, IgE fails to release stored Ca^2+^ when applied following stimulation with thapsigargin (2 μM) in Ca^2+^-free solution. Trace is mean ± SD of 39 cells. *G*, Ca^2+^ oscillations to IgE run down in the presence of the CRAC channel blocker BTP2 (10 μM). *H*, IgE does not raise cytosolic Ca^2+^ when applied after exposure to thapsiargin (2 μM) in 2 mM Ca^2+^-containing solution. Trace is mean ± SD of 30 cells. *I*, comparison of Ba^2+^ entry to IgE with different concentrations of thapsigargin. Cells were stimulated with IgE or different concentrations of thapsigargin in Ca^2+^-free solution before perfusion with 2 mM Ba^2+^. – background denotes basal Ba^2+^ entry rate. Ba^2+^ permeates CRAC channels but is not exported out of the cytoplasm by Ca^2+^ ATPase pumps and therefore provides a reliable measure of ion flux through CRAC channels (49). *J*, aggregate data from experiments as in *panel I*. Each point is a cell, and n >50 for each condition. Data are from three independent experiments. *K*, aggregate data compare the number of Ca^2+^ oscillations over a 15 min recording period for the conditions indicated. Each point is a cell and n = 216 for IgE in 2 Ca^2+^, n = 174 for IgE in 0Ca^2+^, n = 111 for IgE in BTP2, n = 159 fo IgE in U73122 and n = 39 for IgE in U7734. ∗∗ denotes *p* < 0.01 and ∗∗∗ indicates *p* < 0.001, n.s. is not significant. Analyses were carried out using a one way Anova test with posthoc comparison.

To distinguish between the two models, we first characterized the molecular basis of cytosolic Ca^2+^ oscillations in mast cells and the related cell line RBL-2H3. We applied a sub-maximal concentration of IgE (5 μg/ml) to activate endogenous phospholipase Cγ-coupled FCεRI receptors (21) and measured cytosolic Ca^2+^ using the fluorescent indicator Fura-2. In almost all RBL-2H3 (746/821) and mast (124/157) cells, IgE evoked cytosolic Ca^2+^ oscillations (Figs. 2, *A*–*C* and S1, *A* and *B*). The majority of RBL cells (65%) generated numerous cytosolic Ca^2+^ oscillations of similar amplitude that arose from the baseline of resting Ca^2+^ (Fig. 2*A*). In the second type of response (∼15%), several Ca^2+^ oscillations arose but these varied in amplitude (2-5-fold) and occurred on an elevated baseline (Fig. 2*B*). A third pattern (∼10%) was characterized by a few Ca^2+^ oscillations which were followed by a Ca^2+^ plateau (Fig. 2*C*). ∼10% of cells failed to respond to IgE. The Ca^2+^ responses ran down rapidly in the absence of external Ca^2+^ (Fig. 2, *D* and *K*). Readmission of external Ca^2+^ led to a rise in cytosolic Ca^2+^ as Ca^2+^ entered the cell through CRAC channels (Fig. 2*D*). In ∼50% of the cells, Ca^2+^ oscillations started to develop approximately 10 min after Ca^2+^ readmission, whereas a slowly declining non-oscillatory Ca^2+^ signal was seen in the other 50% of cells. Oscillations were suppressed by pre-treatment with the phospholipase C inhibitor U73122, but not its non-active enantiomer U73343 (Figs. 2, *E* and *K* and S1*B*), and were abolished by prior ER store depletion using the sarcoendoplasmic reticulum Ca^2+^-ATPase (SERCA) inhibitor thapsigargin (Figs. 2*F* and S1*C*). Consistent with a role for CRAC channels, Ca^2+^ oscillations ran down in the presence of the CRAC channel blocker BTP2 (Figs. 2, *G* and *K* and S1*A*), in agreement with previous work (21). IgE failed to alter cytosolic Ca^2+^ when applied during thapsigargin treatment in the continuous presence of external Ca^2+^ (Figs. 2, *H* and S1*D*), demonstrating that IgE and thapsigargin activate the same Ca^2+^ entry pathway. However, IgE activated CRAC channels to a lesser extent than strong store depletion with 100 nM thapsigargin (22) (Fig. 2*I*), inducing CRAC channel activity to approximately the same extent as 20 nM thapsigargin (Fig. 2*J*).

**Figure 3:**
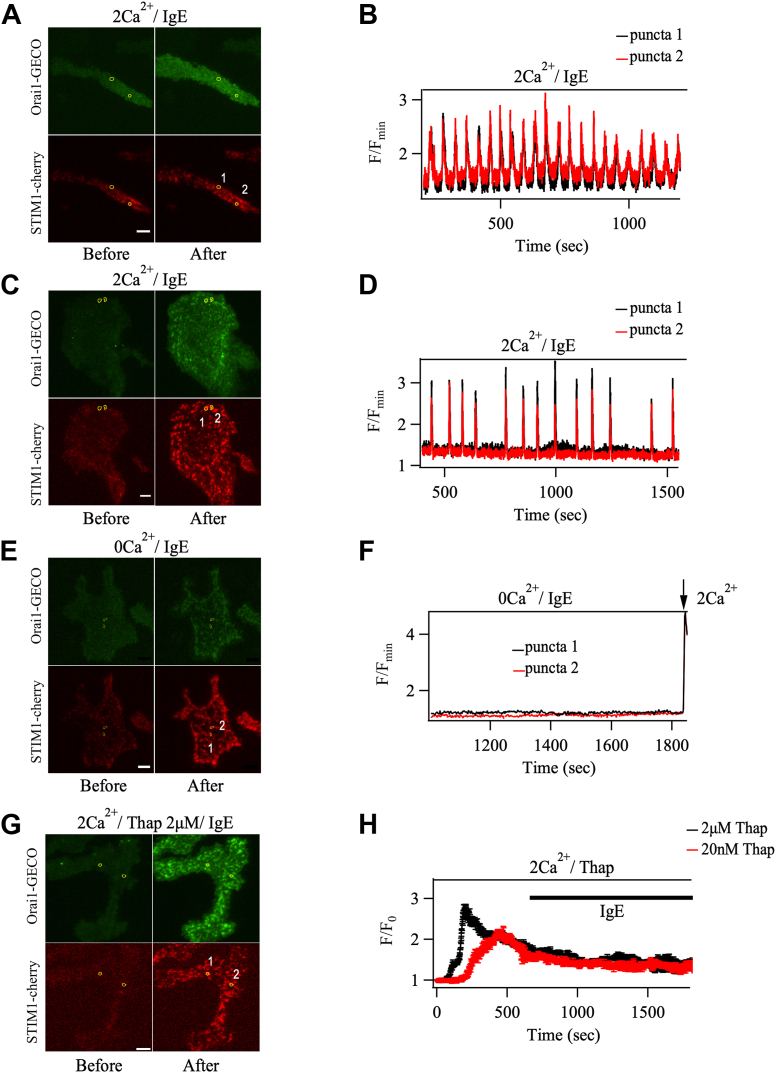
**IgE stimulation leads to oscillatory Orai1-GECO signals in RBL-2H3 cells.***A*, TIRF images (TIRF acquisition/analysis setup 1) show STIM1-cherry and Orai1- GECO distribution before and after stimulation with IgE in 2 mM Ca^2+^- containing solution. *B*, kinetics of Orai1-GECO signals in puncta highlighted in *A*. *C* and *D*, as in *A* but from a different cell and preparation in which selected puncta were close together. *E*, STIM1-cherry and Orai1-GECO localization before and after stimulation with IgE in Ca^2+^-free solution. *F*, Orai1-GECO signals in puncta highlighted in *E*. Responses were absent until external Ca^2+^ was readmitted. *G*, STIM1-cherry and Orai1-GECO localization before and after stimulation with thapsigargin (2 μM) followed by IgE. *H*, 20 nM thapsigargin produced a sustained rise in Orai1- GECO signal in Ca^2+^-containing solution (12 puncta from six cells). The slow decline in the signal reflects Ca^2+^-dependent inactivation of the channels. IgE failed to increase the Orai1- GECO signal after depletion of the ER Ca^2+^ store with 2 μM thapsigargin (10 puncta from five cells). Data are averages±SD. Scale bar denotes 5 μm.

In T cells, low concentrations of thapsigargin evoke cytosolic Ca^2+^ oscillations which are totally dependent on Ca^2+^ entry (20). By contrast, in RBL-2H3 cells, we consistently failed to see cytosolic Ca^2+^ oscillations in response to thapsigargin stimulation at the single cell level, over a range of concentrations from 10 nM to 1 μM (Figs. 2*I*, 3*H*, and S2 for 1 μM). We failed to detect a reliable Ca^2+^ signal with 1 nM thapsigargin. In RBL-2H3 cells, oscillations are therefore a consequence of InsP_3_-dependent Ca^2+^ release followed by store-operated Ca^2+^ entry.

Having established that IgE can stimulate Ca^2+^ release from thapsigargin-sensitive Ca^2+^ stores in mast and RBL cells, and that the store depletion activates Ca^2+^ entry through CRAC channels to sustain the oscillatory Ca^2+^ responses, we sought to measure the temporal dynamics of Ca^2+^ flux through CRAC channels. Orai1-GECO is a genetically encoded protein comprising a fluorescent Ca^2+^ indicator derived from GCaMP3 (GECO) fused to the N-terminus of Orai1, allowing local Ca^2+^ flux through Orai1 channels to be monitored (23, 24). These local fluxes generate Ca^2+^ nanodomains that extend 10 to 20 nm from the mouth of the channel into the cytosol (25). We co-expressed Orai1-GECO with STIM1-cherry in RBL cells (due to their relative ease of transfection) and used total internal reflection fluorescence (TIRF) microscopy (setup 1, see Experimental procedures) to image fluorescence within ∼100 nm of the plasma membrane. Precautions were taken to ensure recombinant proteins were expressed at similar levels to endogenous proteins.

### Orai1 activity fluctuates during oscillatory Ca^2+^ signals

To determine whether CRAC channel activity is sustained or pulsatile during store-operated Ca^2+^ entry, we measured Orai1-GECO signals at individual puncta following stimulation with IgE. Puncta were identified as co-localized clusters of STIM1-cherry and Orai1-GECO that formed after stimulation and were traced as described in Methods. Orai1-GECO signals from a single punctum in RBL cells reflect the activation of endogenous and overexpressed channels and it is estimated that ∼5 to 10 channels are found in a single punctum (26, 27). After stimulation with IgE, STIM1-cherry formed puncta just below the plasma membrane, which led to clustering of Orai1-GECO channels at these sites (Fig. 3, *A* and *C*). Interestingly, Orai1-GECO signals at these presumptive Ca^2+^ influx loci oscillated repetitively throughout IgE stimulation (Fig. 3, *B* and *D*). The number of Orai1-GECO oscillations (9.8 ± 0.9 over 15 min) was very similar to the number of IgE-evoked cytosolic Ca^2+^ oscillations over the same time period (Fig. 2*K*). Stimulation with IgE in Ca^2+^-free solution failed to evoke Orai1-GECO signals (Fig. 3, *E* and *F*), despite formation of both STIM1-cherry puncta and a limited number of large cytosolic Ca^2+^ oscillations (Fig. 2, *D* and *I*). Readmission of external Ca^2+^ rapidly increased the Orai1-GECO signal (Fig. 3*F*) as Ca^2+^ entered through the open CRAC channels. Oscillations of Orai1-GECO in individual puncta were not observed when cells were stimulated with 20 nM thapsigargin in Ca^2+^-containing extracellular solution (Fig. 3, *G* and *H*), a concentration that activated CRAC channels to a similar extent as IgE (Fig. 2*J*). Instead, an increase in local Ca^2+^ was observed that slowly declined (Fig. 3*H*). Furthermore, IgE failed to induce oscillatory Orai1-GECO signals in puncta when applied after 2 μM thapsigargin (Fig. 3*H*), demonstrating that cyclical Ca^2+^ fluxes through Orai1 require repetitive store depletion.

### Orai1 activity fluctuates in phase with cytosolic Ca^2+^

**Figure 4:**
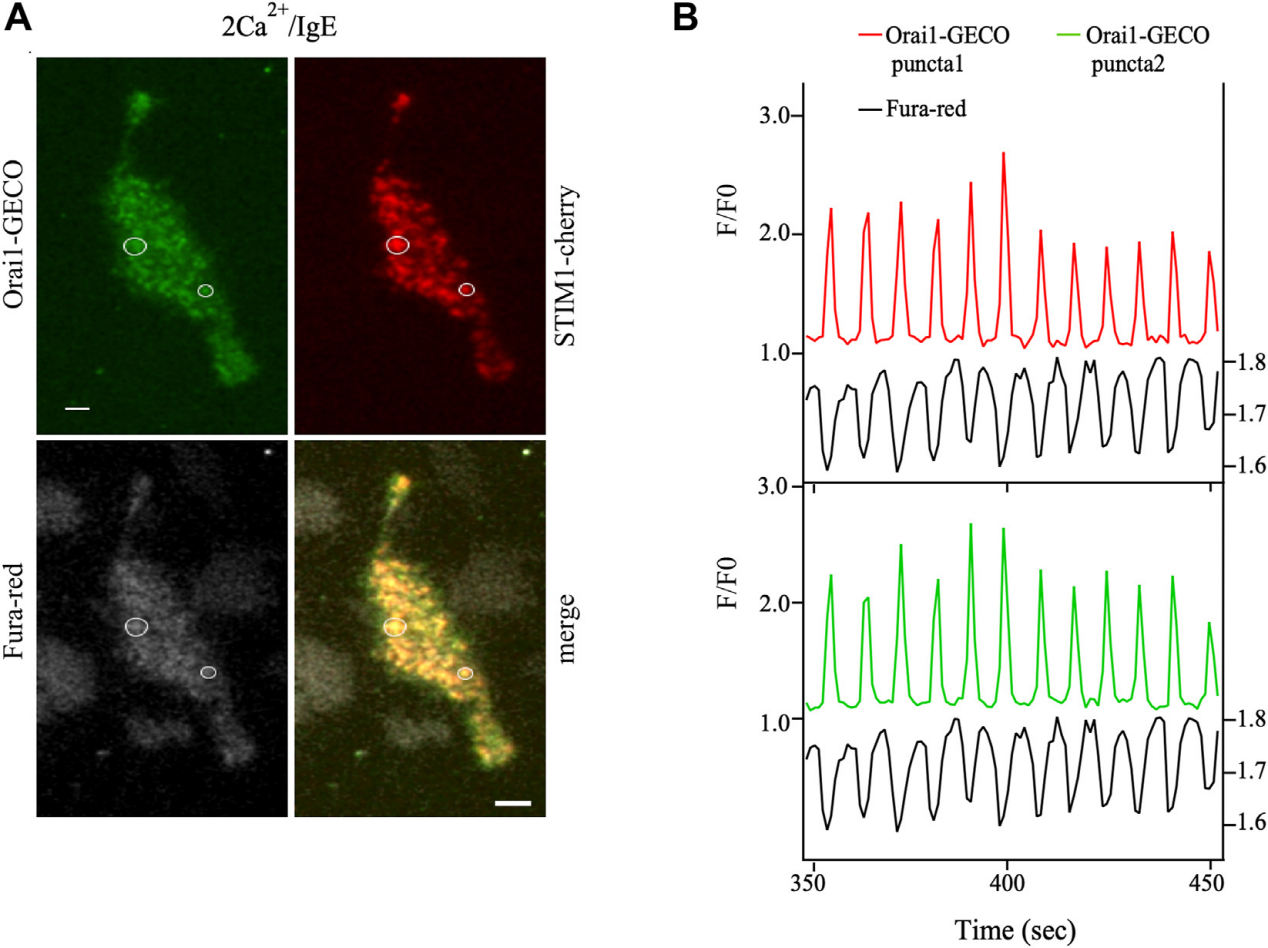
**Orai1-GECO fluctuations correlate with cytosolic Ca^2+^ oscillations.***A*, TIRF images (TIRF acquisition/analysis setup 3) compare Orai1-GECO, STIM1-cherry and Fura Red signals in an RBL cell stimulated with IgE in Ca^2+^-containing extracellular solution. *B*, kinetics of Orai1-GECO and cytosolic Ca^2+^ oscillations for the two puncta shown in (*A*). The Fura Red signal is the same for both puncta as it was taken from the same cell at the same time. Scale bar denotes 5 μm.

We asked whether Ca^2+^ flux through CRAC channels was related to global cytosolic Ca^2+^ oscillations by simultaneously imaging sub-plasmalemmal Orai1-GECO signals and bulk cytosolic Fura Red fluorescence in the same cells. We repeatedly switched between normal TIRF and oblique TIRF (Setup 3; see Experimental procedures) to measure sub-plasmalemmal Ca^2+^ flux through Orai1-GECO and Fura Red-dependent cytosolic Ca^2+^ signals, respectively. IgE stimulation induced formation of STIM1-cherry puncta and co-localization of Orai1-GECO (Fig. 4*A*). Exposure to IgE evoked oscillations in cytosolic Ca^2+^ (Fura Red) which correlated tightly with oscillations in the Orai1-GECO signal (Fig. 4*B*). Correlation analysis between Fura Red and Orai1-GECO responses showed a high degree of association (Pearson correlation value −0.54 ± 0.16; 22 puncta from 10 cells), as would be expected if oscillatory Ca^2+^ release led to transient opening of CRAC channels. Correlation values ranged from −1 to +1 and included negative values because Ca^2+^-sensitive Fura Red signals at 488 nm excitation fell as cytosolic Ca^2+^ rose. These data show that Ca^2+^ flux through Orai1 channels cycles largely in phase with intracellular Ca^2+^ oscillations.

### Orai-GECO measures local Ca^2+^ entry through CRAC channels

**Figure 5:**
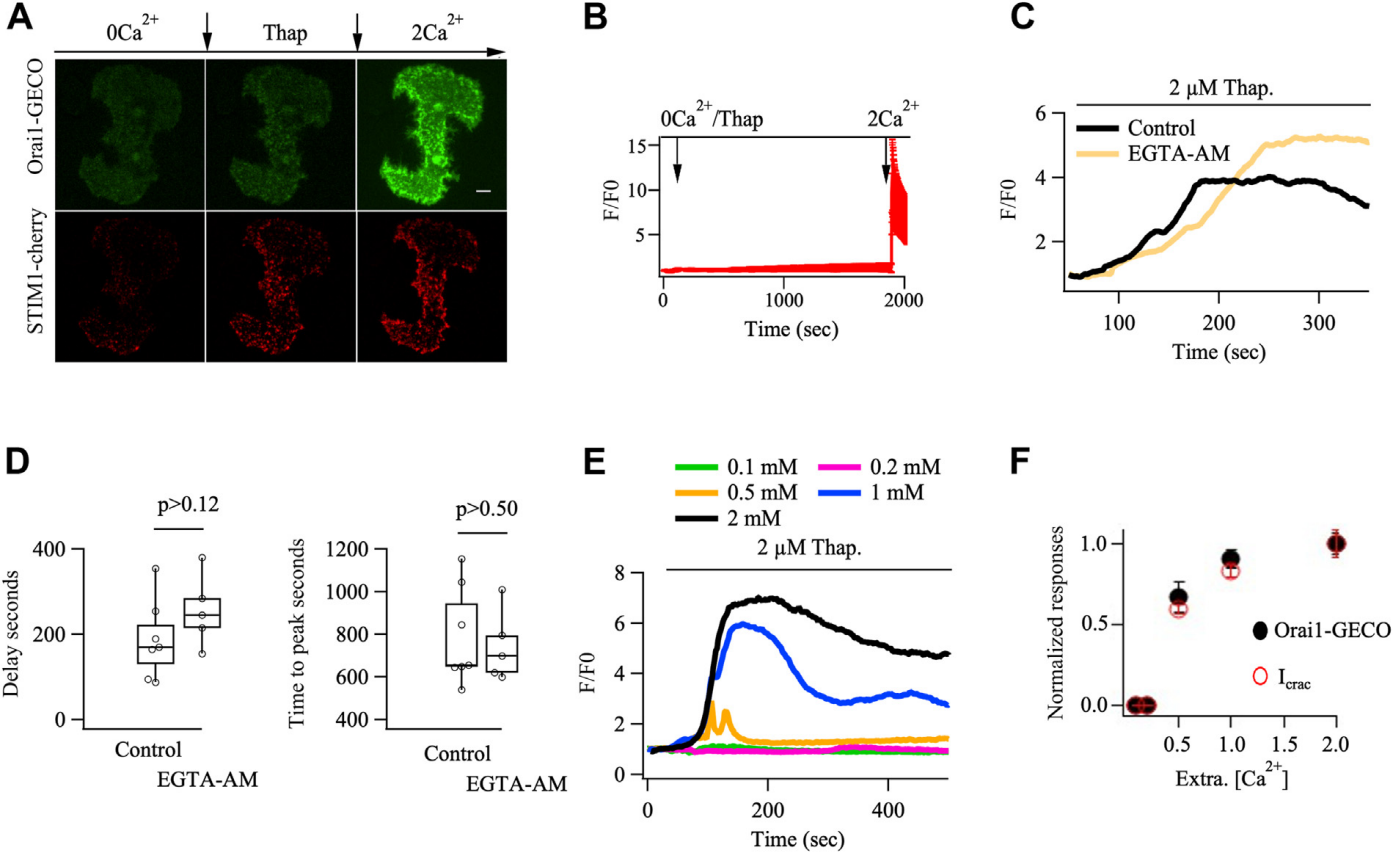
**Validation of Orai1-GECO signals using TIRF microscopy**. *A*, TIRF images (TIRF acquisition setup 1) show distribution of STIM1-cherry and Orai1-GECO in the same RBL-2H3 cell in Ca^2+^-free solution (*left* set of images). Thapsigargin stimulation in Ca^2+^-free solution led to STIM1-cherry clustering into punctate regions within the TiRF field (*middle panel*). In the absence of external Ca^2+^, Orai1-GECO fluorescence was low. Readmission of external Ca^2+^ resulted in a strong Orai1-GECO signal and revealed clusters of Orai1-GECO that mirror those of STIM1-cherry (*right* set of images). *B*, stimulation with thapsigargin (2 μM) in Ca^2+^-free solution failed to generate an Orai1-GECO signal, but a strong response was elicited upon readmission of external Ca^2+^. Trace is mean ± SD of 37 cells. *C*, Orai1-GECO signals are compared for the different conditions indicated for two different cells. In the EGTA-AM experiments, cells were loaded with 10 μM EGTA-AM for 45 min followed by a 15 min de-esterification prior to the onset of recordings. *D*, delay and time to peak from experiments as in *panel C* are compared. Each point is a cell and n = 8 for control and n = 5 for EGTA-AM. *E*, Orai1-GECO signals are compared for cells stimulated with 2 μM thapsigargin in different external Ca^2+^ concentrations. Each trace is a separate cell. *F*, Graph compares the peak Orai1-GECO signal (from experiments as in *panel E*) with the size of I_CRAC_ in the presence of different external Ca^2+^ concentrations. Patch clamp data were taken from ref. 32. Scale bar denotes 5 μm.

**Figure 6:**
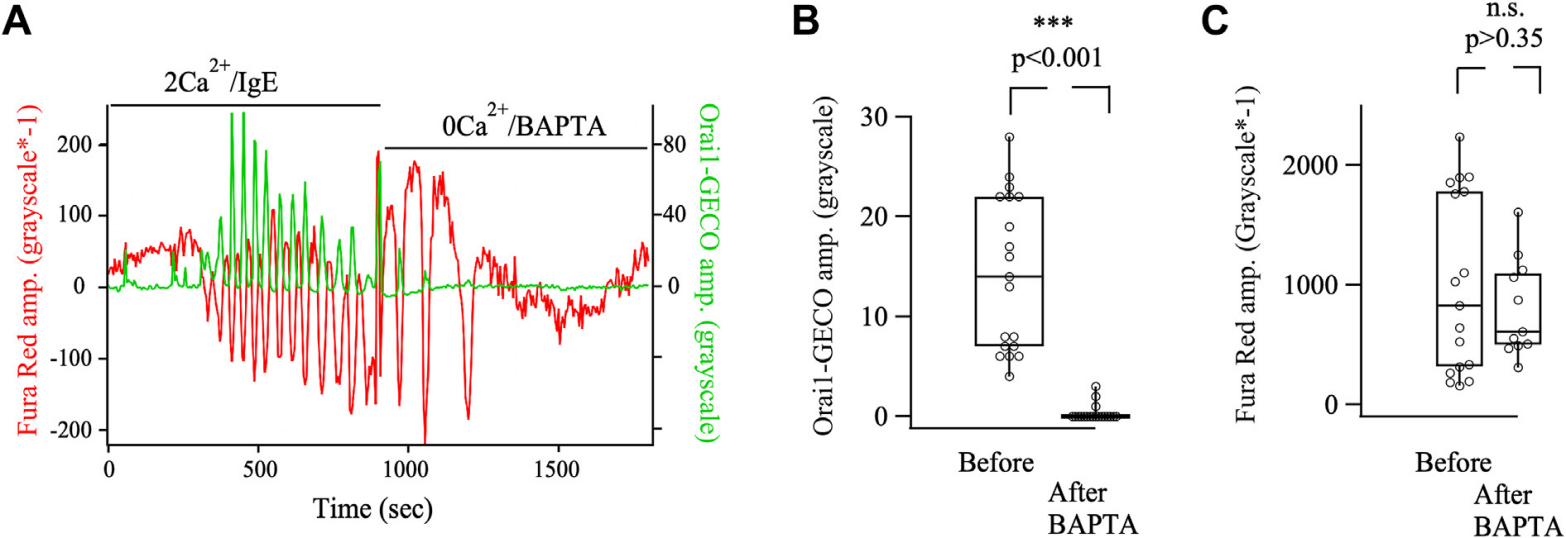
**Orai1-GECO faithfully tracks local Ca^2+^ influx**. *A*, Orai1-GECO (*green trace*) and Fura red (*red trace*) fluorescence before and after IgE stimulation were measured in the same RBL-2H3 cell using TIRF (TIRF acquisition/analysis setup 3). IgE was applied in 2 mM Ca^2+^-containing extracellular solution. Ca^2+^-free solution was supplemented with 0.5 mM BAPTA to rapidly remove extracellular Ca^2+^. Orai1-GECO signals disappeared quickly, whereas large amplitude cytosolic Fura red responses were maintained for ∼ 180 s. *B*, box plot compares amplitude of Orai1-GECO signals for two spikes just before and then after removal of extracellular Ca^2+^ (labelled BAPTA). *C*, box plot shows the corresponding Fura red signals just before and then after removal of extracellular Ca^2+^. In some experiments, only one Ca^2+^ spike occurred after switching to Ca^2+^-free solution. Each point is a single cell, ∗∗∗ denotes *p* < 0.001 and n.s. not significant based on unpaired Student's *t* test.

To validate Orai1-GECO as a reliable measure of local Ca^2+^ entry through Orai1 channels, we conducted a series of control experiments. First, we stimulated cells with thapsigargin in the absence of external Ca^2+^. Store depletion resulted in formation of STIM1 puncta accompanied by co-localization of Orai1 channels (Fig. 5*A*), but no detectable increase in Orai1-GECO signal was detected within the TiRF field (Fig. 5*B*). When external Ca^2+^ was readmitted, a large Ca^2+^ signal now occurred (Fig. 5, *A* and *B*). Second, as reported previously (28), the Orai1-GECO signal was abolished when BTP2 was applied to cells stimulated with thapsigargin in the presence of extracellular Ca^2+^. Third, if the Orai1-GECO signals are confined to the TiRF field, a prediction is that loading the cytosol with the slow Ca^2+^ chelator EGTA should not reduce the size or kinetics of the local Ca^2+^ signal. EGTA is too slow to capture Ca^2+^ as it enters the cytosol and therefore cannot buffer Ca^2+^ within ∼120 nm of the channel pore but the chelator suppresses the global Ca^2+^ rise (25, 29). Loading the cytosol with EGTA neither reduced the size (Fig. 5*C*) nor the time course of the Orai1-GECO signal (Fig. 5*D*). If the Orai1-GECO signal was a consequence of Ca^2+^ diffusion from the cytosol to the channels, cytosolic EGTA would have slowed the development of the Orai1-GECO signal, delaying the rise and increasing the time to peak. The Orai1-GECO signal was more prolonged in cytosolic EGTA at later times, reflecting a reduction in Ca^2+^-dependent slow inactivation of the channels, which requires a rise in bulk Ca^2+^ (30, 31). Fourth, if the GECO signal reflects local Ca^2+^ near open Orai1 channels, it should mirror the unitary flux through the channels. In RBL cells, I_CRAC_ scales linearly with external Ca^2+^ over the range 0.1 to 2 mM, with an effective K_D_ for permeation of 0.7 mM (32). If the Orai1-GECO signal faithfully monitors local Ca^2+^ entry, then the fluorescence should also scale linearly over the range 0.1 to 2 mM external Ca^2+^. Orai1-GECO signals in different external Ca^2+^ concentrations are compared in Figure 5*E*, and these superimposed with the external Ca^2+^-dependence of I_CRAC_ (Fig. 5*F*). Finally, we exploited the fact that once cytosolic Ca^2+^ oscillations have been induced by IgE in Ca^2+^-containing solution, removal of external Ca^2+^ rapidly abolishes Ca^2+^ entry but regenerative Ca^2+^ release from the stores continues and generates at least 2 to 3 oscillations. These Ca^2+^ spikes in Ca^2+^-free solution are large and more prolonged, likely a consequence of loss of Ca^2+^-dependent inhibition of InsP_3_ receptors by Ca^2+^ entry. We tracked Orai1-GECO signals simultaneously with measurements of cytosolic Ca^2+^ using Fura-Red. Stimulation with IgE led to oscillations in Orai1-GECO (green trace in Fig. 6*A*) that were closely aligned with oscillations in cytosolic Ca^2+^ (red trace in Fig. 6*A*). Exposure to Ca^2+^-free solution supplemented with BAPTA to rapidly chelate extracellular Ca^2+^ resulted in almost immediate cessation of the Orai1-GECO signal, but three robust cytosolic Ca^2+^ transients still occurred which were slightly larger than the preceding oscillations in the presence of extracellular Ca^2+^(Fig. 6*A*). We measured the amplitude of the 1 to 2 spikes just before and then immediately after removal of external Ca^2+^. While the Orai1-GECO signal was abolished (Fig. 6*B*), the Fura-red signal was still robust (Fig. 6*C*). Collectively, these data demonstrate that Orai1-GECO provides a reliable measure of local Ca^2+^ flux through open CRAC channels (23, 24) and the signals are unaffected by large Ca^2+^ signals arising from regenerative Ca^2+^ release.

### Spatially distinct Orai1 clusters cycle in phase

**Figure 7:**
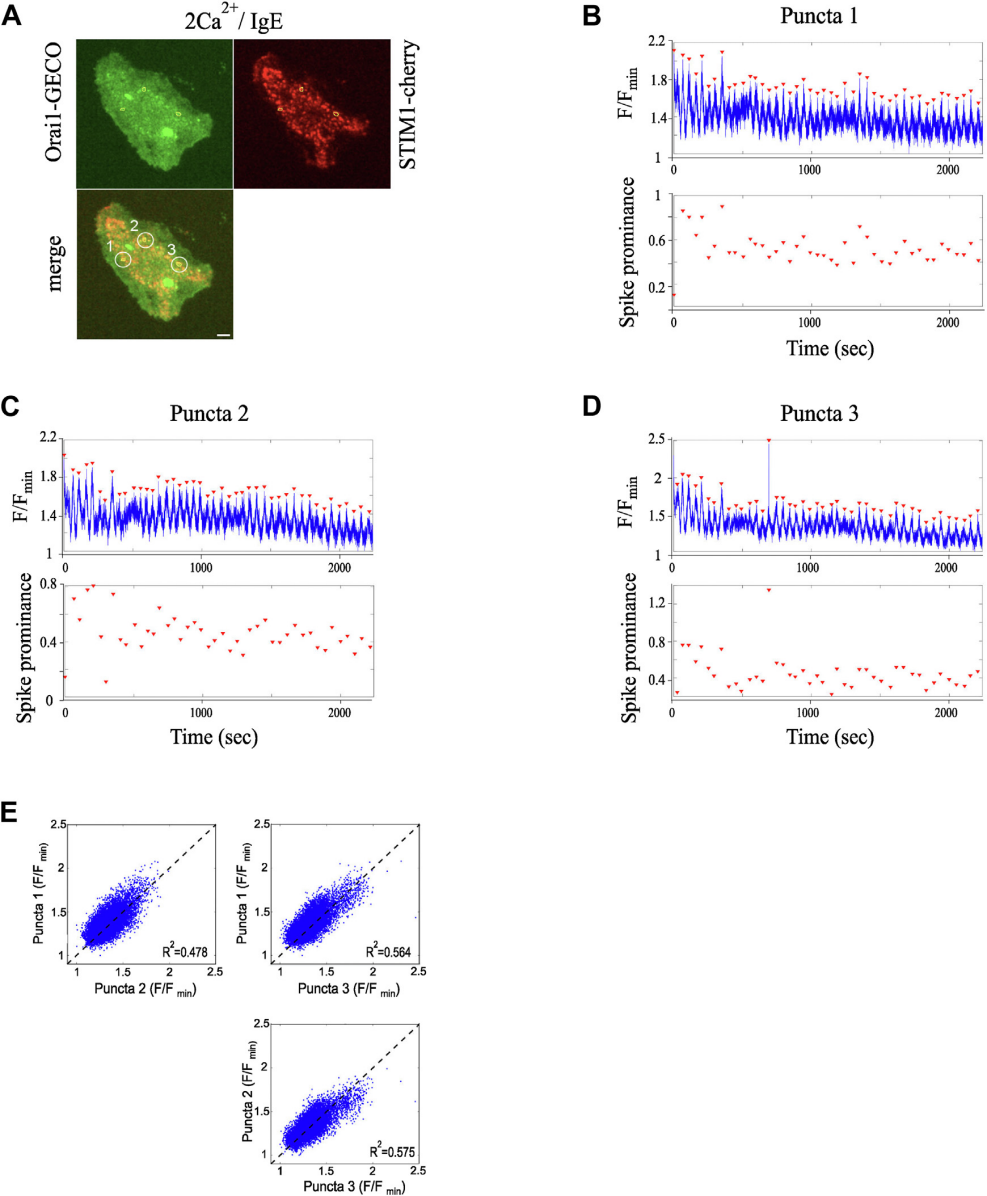
**Orai1-GECO signals in different puncta**. *A*, TIRF images (TIRF acquisition/analysis setup 2) show Orai1-GECO and STIM1-cherry distribution after stimulation with IgE in 2 mM Ca^2+^. Three puncta (1–3) were stable over a ∼35-min recording period. *B*, Orai1-GECO signals (*upper panel*) and oscillatory amplitudes (*lower panel*) are shown for punctum 1 over time. *C* and *D*, as in *B* but for punctum 2 and 3, respectively. *E*, correlation plots for puncta 1, 2, and 3 are compared. The *black dashed lines* are 1:1 lines to indicate what a perfect correlation would look like. All data were from RBL-2H3 cells. Scale bar denotes 5 μm.

We noticed that the amplitude of Orai1-GECO oscillations gradually decreased over time in the majority of puncta (for example, see Figs. 3*B* and 4*B*). This could reflect Ca^2+^-dependent inactivation of Orai channels or photobleaching of the probe. Because the signals still decayed when acquisition rates were reduced from 200 ms to 5 s (Fig. 7, *A* and *B*; TIRF setup 2, see Experimental procedures), we surmised that the decline reflected a decrease in Ca^2+^ flux through the channels.

Despite this rundown, fluctuations in Orai1-GECO puncta were sufficiently robust that different puncta within the same cells could be analyzed simultaneously. An example of a cell in which we recorded three active puncta is shown in Figure 7*A*. Throughout the 1000 to 1500 s recording period, Ca^2+^ signals tended to repeat at regular intervals (Fig. 7, *B*–*D*). Different puncta cycled largely in phase, peaking at the same times, although signal amplitudes varied between puncta (Fig. 7, *B*–*D*). For each punctum, the amplitude of the Orai1-GECO signal fell with time, reaching a steady state that was ∼60% of the initial peak size (Fig. 7, *B*–*D*). The correlation between Orai1-GECO peaks in different puncta was reasonably good, with R-squared (square of the correlation) values around 0.5 (Fig. 7*E*). These correlations were independent of proximity between puncta. Similar findings were made in eight other cells in which with 2 to 3 puncta were analyzed, confirming that individual puncta in single cells cycle in phase, independent of their relative locations.

## Discussion

Two key findings arise from our study. First, CRAC channel activity fluctuates in close temporal synchrony with bulk cytosolic Ca^2+^ oscillations. Second, CRAC channels in puncta located some distance apart nevertheless fire in phase with one another.

InsP_3_-driven cytosolic Ca^2+^ oscillations are sustained by Ca^2+^ influx through CRAC channels (10). However, the question of whether CRAC channel activity remains constant during stimulation or fluctuates with Ca^2+^ oscillations has been a subject of debate since the 1990s. One model proposes that CRAC channels remain open throughout the stimulatory period, providing a continuous flux of Ca^2+^ that functions as a pacemaker in setting oscillation frequency (16, 17, 18). An alternative model posits that CRAC channels provide bursts of Ca^2+^ entry because the channels oscillate in phase with agonist-evoked cyclical Ca^2+^ release from the ER (19, 20).

Our new data provide direct evidence in support of the latter model, wherein CRAC channels pulsate in phase with InsP_3_-dependent regenerative Ca^2+^ release from the ER. Resolution of this issue is important because it provides new insight into how Ca^2+^ influx is decoded by cells to drive downstream responses. CRAC channels participate in a signalosome with the scaffolding protein AKAP79 (33, 34, 35). AKAP79 binds various signaling molecules including calmodulin (36), protein kinases A and C and the Ca^2+^-activated protein phosphatase calcineurin (37), and the transcription factor NFAT (38, 39). Ca^2+^ nanodomains near open CRAC channels stimulate calcineurin, which dephosphorylates and activates NFAT (33, 40). NFAT then translocates to the nucleus to regulate expression of a variety of chemokines and cytokines that help shape an inflammatory response. Pulsatile CRAC channel activity would enable both amplitude and frequency-dependent activation of these downstream targets. The frequency of cytosolic Ca^2+^ oscillations arising from InsP_3_-driven regenerative Ca^2+^ release from the ER increases with agonist concentration (1). Because oscillatory Ca^2+^ release is largely in phase with CRAC channel fluctuations, the frequency of pulsatile Ca^2+^ entry through CRAC channels should show an identical dependence on agonist concentration. The phase synchronization between global cytosolic Ca^2+^ oscillations and pulsatile CRAC channel activity provides a mechanism for coordinating the activities of signaling molecules across two distinct spatial domains—the cytosol and cell periphery—ensuring their simultaneous activation within the same temporal framework.

Different agonists evoke different patterns of cytosolic Ca^2+^ oscillation, even in the same cell type, and which arise from different mechanisms (41). In pancreatic acinar cells for example, cytosolic Ca^2+^ oscillations following stimulation of G protein-coupled cholecystokinin receptors are sustained for several minutes in Ca^2+^-free extracellular solution (42), in sharp contrast to our experiments with IgE. Nevertheless, despite these caveats, several agonists generate cytosolic Ca^2+^ oscillations through InsP_3_-dependent Ca^2+^ release from the endoplasmic reticulum, which require Ca^2+^ entry through CRAC channels to refill the Ca^2+^ store. We therefore think our findings are of relevance to this common form of cytosolic Ca^2+^ oscillation.

Multiple clusters of Orai1 display synchronized activity, despite some being spatially remote from others. Thus, in contrast to a channel crosstalk mechanism, Orai1 synchronization likely reflects rapid depletion of peripheral ER during stimulation. Peripheral ER is closely opposed to the plasma membrane, forming ER-plasma membrane junctions (43). These junctions are typically 200 nm in length with the ER component lying within ∼ 17 nm of the plasma membrane (44), a distance that permits direct binding between STIM proteins and Orai1. The junctions occupy as much as 4% of the plasma membrane, depending on cell type (26). Immobilized InsP_3_ receptors cluster at ER-PM junctions and rapidly deplete peripheral ER of Ca^2+^ (45). Having swathes of ER-PM junctions populated with InsP_3_ receptors therefore provides a mechanism for ensuring CRAC channel puncta that are located far apart nevertheless activate together, following rapid release of Ca^2+^ from peripheral ER. It was not possible to measure the activities of all Orai1 clusters in the TiRF field at the same time because many puncta in live cell imaging moved in and out of focus and therefore were not stable enough for us to record from them continuously for tens of minutes. Moreover, the TiRF measurements are confined to a limited section of the cell periphery. Therefore, we cannot extrapolate our findings from a few stable clusters to all clusters across the cell. Nevertheless, the finding that several CRAC channel clusters fire together suggests that downstream responses could be dictated by summation of individual clusters, analogous to the motor end plate potential at the neuromuscular junction, which is a function of the number of miniature end plate potentials (46).

## Experimental procedures

### Isolation of bone marrow-derived mast cells

Bone marrow-derived mast cells were isolated from the femurs of C57BL.6 adult mice (47). Briefly, mice (8–12 weeks old) were euthanized by CO_2_ followed by cervical dislocation, and long bones (femur and tibia) removed whilst taking care to keep the bones intact. Under sterile conditions, both ends of isolated bones were removed with scissors, and each bone flushed with 5 ml of Dulbecco's phosphate-buffered saline (DPBS). Bones were then crushed and flushed further. The flushed medium containing a cell suspension was collected, passed through a 70-μm filter, and then centrifuged for 5 min at 800 rpm. The resulting cell pellet was resuspended in Dulbecco's minimum essential medium (DMEM) supplemented with 10% FBS, 1% penicillin/streptomycin and transferred on to 10 cm tissue culture dishes. The cells were cultured at 37 °C in an incubator containing 5% CO_2_ in DMEM supplemented with 10 ng/ml interleukin-3, 10% FBS and 1% penicillin/streptomycin. After 4 weeks differentiation, the percentage of mast cells was >95 mast cells, as measured by flow cytometry of FcεRI receptor expression.

### Cell culture

RBL-2H3 cells (ATCC, CRL 2256) were cultured in DMEM supplemented with 10% heat-inactivated fetal bovine serum, 2 mM glutamine and 1% penicillin/streptomycin, and maintained in a humidified 95% air, 5% CO_2_ incubator at 37 °C (33, 48). Cells were checked for *mycoplasma* infection and were negative.

In preparation for cytosolic Ca^2+^measurements or confocal microscopy, bone narrow-derived and RBL-2H3 cells were sub-cultured onto 30-mm round glass coverslips (#1.5 thickness) and maintained in culture for an additional 36-48 h before use in cytosolic Ca^2+^ measurements.

### Cell transfection

RBL-2H3 cells were transfected using the Amaxa electroporation system. cDNA for STIM1-RFP was a gift from Dr J. Putney (NIEHS), Orai1-GECO was from Addgene. Low levels of plasmid were used (0.1 μg Orai1-GECO and 0.2 μg STIM1-RFP) to minimize overexpression. In one set of experiments, we knocked down endogenous Orai1 before expressing Orai1-GECO. Because similar kinetics of Orai1-GECO signals were obtained to IgE, we did not routinely knock down Orai1.

### Cytosolic Ca^2+^ measurements

Fluorescence measurements were made after loading cells with the Ca^2+^- sensitive dye, fura 2, as described previously (48). Briefly, cells plated on 30 mm round coverslips and mounted in a Teflon chamber were incubated in HEPES- buffered salt solution (composition below) with 1 μM acetoxymethyl ester of fura 2 (Fura-2/AM, Molecular Probes) at room temperature in the dark for 40 min. For cytosolic Ca^2+^ measurements, cells were bathed in HEPES-buffered salt solution (HBSS: NaCl 145; KCl 2.8; MgCl_2_ 2; HEPES 10; CaCl_2_ 2 and glucose 10 mM, with pH 7.4 adjusted by NaOH) at room temperature. Ca^2+^-free solution was HBSS with no added CaCl_2_ and supplemented with 0.1 mM BAPTA. Fluorescence images of the cells were recorded and analyzed with a digital fluorescence imaging system (InCyt Im2, Intracellular Imaging Inc). Fura two fluorescence was monitored by alternatively exciting the dye at 340 and 380 nm and collecting the emission wavelength at 520 nm. Changes in cytosolic Ca^2+^ are expressed as the “Ratio (F340/F380)”. Before starting each experiment, regions of interest identifying cells were created and 10 to 25 cells were monitored per experiment. As needed, ratio values were corrected for contributions by autofluorescence, which was measured after treating cells with 10 μM ionomycin and 20 mM MnCl_2_.

### Total internal reflection fluorescence (TIRF) microscopy

(1) Two color TIRF experiments of STIM1-cherry, and Orai1-GECO (Setup 1) Fluorescence images were captured on an Andor Dragonfly 505 multi modal confocal system (Oxford Instruments) in Total Internal Reflection Fluorescence (TIRF) mode using a Nikon 60X ApoTIRF 60X/1.49 objective lens. The 488 nm laser line was set to 3%, TIRF mode to penetration with a depth of 100 nm while fluorescence emission was collected through a 521/38 filter with a 400 ms exposure of an Andor Zyla camera. Sequentially, the 561 nm laser line was set to 2%, TIRF penetration to 100 nm, while florescence emission was collected through a 594/43 filter with a 200 ms exposure of an Andor iXon camera. Time series images were acquired every 2 s during each experiment. The time series images were analyzed in Bitplane Imaris (version 9.9) where individual STIM1-mCherry puncta were identified and tracked as an Imaris surface over time where the mean fluorescence intensity of the ORAI1-GECCO signal for that punctum was recorded for each timeframe. Only those puncta where STIM1 and Orai1- GECO were stable for > 600 s and did not drift across the boundary of the ROI, were analyzed. These stringent criteria restricted the number of puncta anayzed per cell to 2 to 3.
(2) two color TIRF experiments of STIM1-cherry, and Orai1-GECCO with fast acquisition of Orai1-GECO (Setup 2)

A 10-minute time series acquisition of STIM1-mCherry and Orai1-GECCO were first obtained as described above. After completion of this first time series, a new single-channel TIRF acquisition of only Orai1-GECCO was initiated with the following parameters: The 488 nm laser line was set to 3%, TIRF mode to penetration with a depth of 100 nm while fluorescence emission was collected through a 521/38 filter with a 100 ms exposure of an Andor iXon camera. Images were acquired every 200 ms for a total of 10,000 frames. After completion of this time series then a single two-channel TIRF image of STIM1-mCherry and Orai1-GECO was acquired using the initial settings for confirmation of STIM1 and Orai1 puncta. The images were brought into FIJI (1.54f) where colocalized STIM1 and Orai1 puncta were identified from the first time series, ROIs were stored, and then the ROIs were translated to the high frame rate single channel Orai1-GECO time series. Mean Intensity of these ROIs were measured across the high frame rate time series.

(3) three color TIRF experiments of STIM1-cherry, Orai1-GECCO, and Fura-Red (Setup 3)

Fluorescence images were captured on an Andor Dragonfly 505 multi modal confocal system (Oxford Instruments, Abingdon, UK) in Total Internal Reflection Fluorescence (TIRF) mode using a Nikon 60X Apo TIRF 60X/1.49 objective lens. The 488 nm laser line was set to 3%, TIRF mode to penetration with a depth of 100 nm while fluorescence emission was collected through a 521/38 filter with a 400 ms exposure of an Andor Zyla camera. Sequentially, the 561 nm laser line was set to 2% for STIM-cherry, TIRF penetration to 100 nm, while florescence emission was collected through a 594/43 filter with a 200 ms exposure of an Andor iXon camera. A third channel was then used to image cytosolic calcium by exciting Fura Red through oblique illumination TIRF. The 488 laser (the Ca^2+^-sensitive excitation for Fura Red) was set to 2% and TIRF mode to HiLo with an illumination angle of 5% which places the excitation light well into the cytosol of the cell. The Ca^2+^-sensitive Fura Red emission, which falls as cytosolic Ca^2+^ rises, was collected through a 698/77 filter to Andor iXon camera with an exposure of 250 ms. The experimental protocol was setup to acquire a three channel time series (Orai1-GECO 100 nm penetration TIRF, STIM1-cherry 100 nm penetration TIRF, Fura Red 5° HiLo TIRF) every 5 s for 1000 frames.

No detectable spectral overlap in emission was seen between Fura Red and Orai1-GECO or STIM1-cherry. When cells, loaded with Fura Red and challenged with 2 μM thapsigargin in Ca^2+^-containing external solution, were excited at 488 nm, ∼no emission signal was detected at 521 nm, the wavelength used for detecting GECO signals. The emission at 521 nm was <2% that seen 698 nm, the wavelength used for Fura Red. Similarly, there was no excitation of Fura Red at 561 nm, nor emission at 594 nm when excited at 488 nm, which are the relevant wavelengths for STIM1-cherry.

The time series images were analyzed in Bitplane Imaris (version 9.9) where individual STIM1-mCherry puncta were identified and tracked as an Imaris surface over time where the mean fluorescence intensity of the ORAI1-GECO signal for that punctum was recorded for each timeframe. Additionally, another Imaris surface was created for the full volume of each cell where the Fura Red fluorescence was recorded for the entire cell for each timeframe.

### Puncta correlation analysis

For Figure 7, *B*–*D* the red triangles indicating peaks of F/F_min_ were detected using the MatLab R2023b findpeaks algorithm with a minimum prominence of 0.1 and minimum separation of 35 s. The “prominence” is defined as the height above neighboring troughs, rather than an absolute F/F_min_ value. Scatterplots of F/F_min_ for different puncta in the same cell are shown in Figure 7*E* for the whole time trace. R^2^ value is the adjusted coefficient of determination (the proportion of variance in the variable plotted on the y-axis that is explained by a linear relationship with the x-axis value), which was calculated using MatLab's fitlm method.

## Data availability

All data are contained within the manuscript.

## Supporting information

This article contains supporting information.

## Conflict of interest

The authors declare that they have no conflicts of interest with the contents of this article.
