# Supplementary material for: CRAC channel activity pulsates during cytosolic Ca2+ oscillations
Source: J Biol Chem. 2025 Apr 23;301(6):108519. doi: 10.1016/j.jbc.2025.108519 (PMC12151228; doi:10.1016/j.jbc.2025.108519)
Supplement: Supporting information [file mmc1.zip › SI.docx]

**Supporting Information**

Supplementary Figure 1. IgE stimulation generates cytosolic Ca^2+^ oscillations in bone marrow-derived mast cells. A, Challenge with IgE in 2 mM Ca^2+^-containing solution evoked Ca^2+^ oscillations, which were abolished by addition of the CRAC channel blocker BTP2 (10 μM). Similar results were obtained in 26 cells from 2 biological replicates. B, Ca^2+^ oscillations were abolished by the phospholipase C inhibitor U73122 but not by the inactive analogue (U73343). Identical results were seen in another 37 cells. C, IgE failed to increase cytosolic Ca^2+^ when applied after stimulation with thapsigargin (2 μM) in Ca^2+^-free solution. Trace is the mean+/-SEM of 41 cells. D, IgE does not increase cytosolic Ca^2+^ when applied after stimulation with thapsigargin in Ca^2+^-containing solution. Trace is the mean +/- SEM of 22 cells.

Supplementary Figure 2. Stimulation with thapsigargin does not evoke cytosolic Ca^2+^ oscillations in RBL cells. A-D, Cells were challenged with 10 nM (A), 20 nM (B), 50 nM (C) and 100 nM thapsigargin (D) in 2 mM Ca^2+^-containing extracellular solution. 5 cells are shown for each condition. Data are representative of > 40 cells per condition.
