# Supplementary figures and images for: CRAC channel activity pulsates during cytosolic Ca2+ oscillations
Source: J Biol Chem. 2025 Apr 23;301(6):108519. doi: 10.1016/j.jbc.2025.108519 (PMC12151228; doi:10.1016/j.jbc.2025.108519)

Supplementary Figure 1.

A

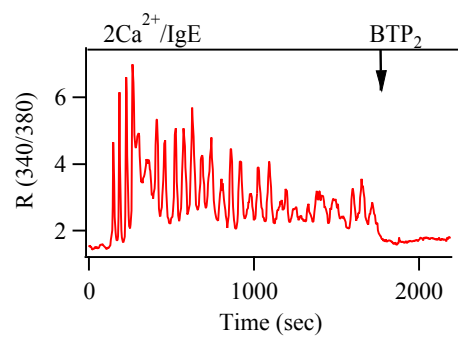

B

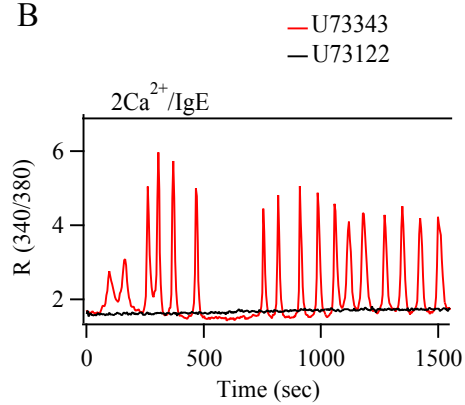

C

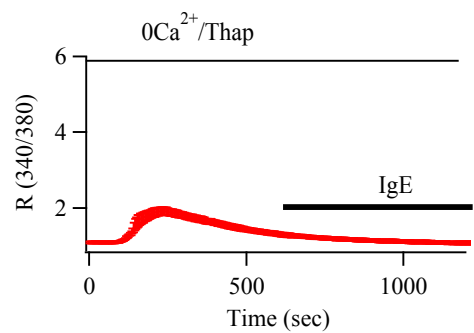

D

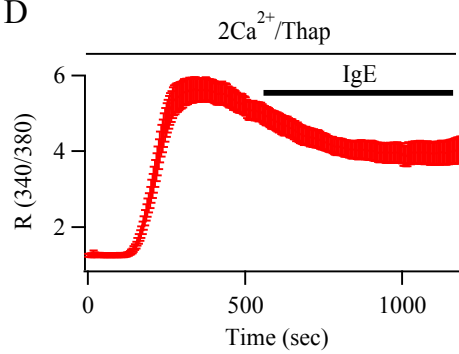

Supplement: Supporting information [file mmc1.zip › Supplementray Figure 1.pdf]

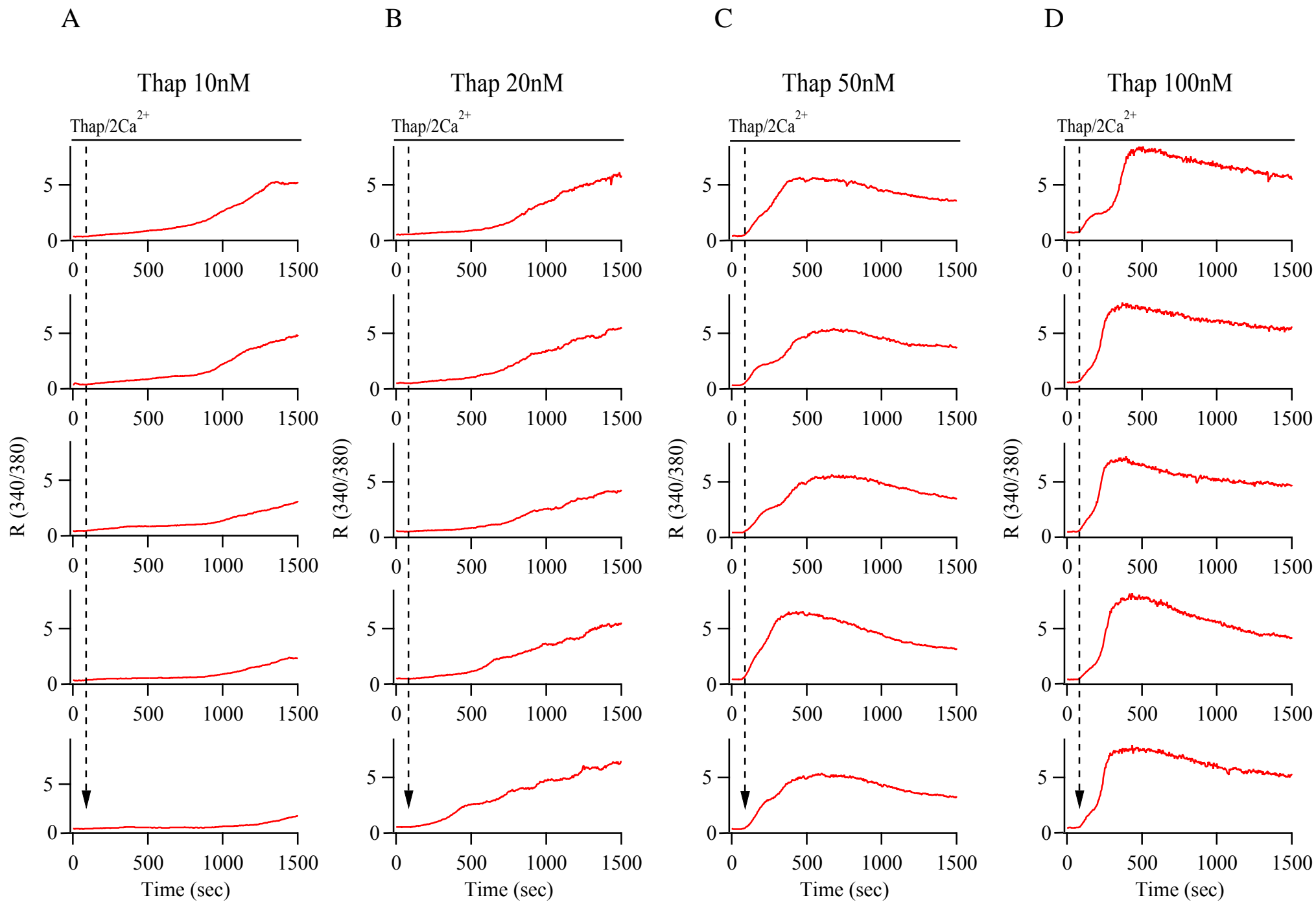

Supplement: Supporting information [file mmc1.zip › Supplementary Figure 2.pdf]
